# Longitudinal Effects of the Pandemic and Confinement on the Anxiety Levels of a Sample of Spanish Children in Primary Education

**DOI:** 10.3390/ijerph182413063

**Published:** 2021-12-10

**Authors:** Marta Giménez-Dasí, Laura Quintanilla, Marta Fernández-Sánchez

**Affiliations:** 1 Faculty of Psychology, Complutense University of Madrid, 28223 Madrid, Spain; 2 Faculty of Psychology, Universidad Nacional de Educación a Distancia (UNED), 28040 Madrid, Spain; lquintanilla@psi.uned.es; 3 Educational Guidance Service, Department of Education of Madrid, 28223 Madrid, Spain; marta.fernandezsanchez1@educa.madrid.org

**Keywords:** anxiety, COVID-19, primary education children, longitudinal study

## Abstract

(1) Background: The psychological effects of confinement due to the SARS-CoV-2 virus pandemic on children are only partially known. In Madrid, Spain, children suffered a strict confinement for 10 weeks and they returned to school under conditions that were far from normal. This work assesses the effects of the pandemic on the anxiety levels of a group of children living in Madrid. (2) Methods: Children were aged 6 to 11 years (N = 215). A self-report measure of anxiety was completed by participants at two time-points: (1) a few months before the beginning of the pandemic and (2) 1 year later. A smaller subgroup of participants also completed the measure during the confinement period (*n* = 60). (3) Results: A comparison of these three measures shows that the children’s anxiety was reduced during confinement, and that one year later these levels continue below those registered before the start of the pandemic. (4) Conclusions: These results contradict some previous studies, which found an increase in children’s anxiety as a result of confinement and the pandemic. The discussion considers protective and vulnerability factors in the context of the pandemic, which may affect children’s levels of anxiety.

## 1. Introduction

The health emergency generated by the Severe Acute Respiratory Syndrome Coronavirus 2 (SARS-CoV-2) is affecting our society in many different ways. Home confinement was one of the most drastic measures ever adopted to contain the propagation of a pandemic in countries around the world. The duration and characteristics of this confinement have varied according to the country and the incidence of the disease. In Madrid, Spain, the declaration of a state of emergency on 14 March 2020 enforced a total confinement of the population for 6 weeks, during which the schools were shut. At the end of April 2020, the measures began to be gradually eased, allowing children to go out of their homes for 1 h a day, although the schools remained closed. These measures remained in force for a further month. Children, therefore, remained confined or in a system of partial isolation for a total of 10 weeks. Later, the return to school in September was carried out in conditions that were very different to normal. Schools have reduced the number of children per classroom; they have established student bubbles, limited contact between peers, eliminated the activities that involve physical contact, reduced spaces for play and obliged the children to wear masks at all times. All these measures have resulted in the children being greatly limited in their normal play and extra-curricular activities, as well as in their contact with other children.

### 1.1. Psychological Effects of the Pandemic on Children

Previous research from other pandemic disasters that have included disease-containment measures, such as quarantine and isolation, have already found that the risk of suffering from post-traumatic stress increases by up to four times in children and adolescents who had been confined compared to those who had not had this experience [1]. In the specific case of the COVD-19 pandemic, there are also studies that have pointed to some negative consequences of confinement among children. For example, a significant increase in anxiety and depression was observed in a study based on parent reports carried out in a sample of 2330 children aged between 7 and 12 years who were confined in China [2]. Similarly, another study also found that the parents of confined children aged between 3 and 18 years observed an increase in irritability, increased rates of distraction and greater clinginess [3].

The first European study on the effects of confinement on the child population, carried out through an online survey during confinement on 1143 parents of Italian and Spanish children aged 3 to 18 years, found greater emotional and conduct problems among Spanish than Italian children, with more difficulties in concentration, greater irritability, boredom, loneliness and unease [4]. The same authors compared parents’ reports of Spanish, Italian and Portuguese children aged 3 to 18 years at different time-points during confinement. The results showed an increase in anxiety between weeks 2 and 5 of confinement and a decrease between weeks 5 and 8 of confinement [5]. Another study found that the parents of Spanish children aged 3 to 12 years reported negative reactions in 69% of children, sleep problems in 31% and conduct problems in 24% [6]. More recently, a study on the effects of confinement on emotional wellbeing in a sample of Spanish children aged from 3 to 11 years also found that parents reported a significant increase in problems relating to emotion regulation, attention, hyperactivity and impulsiveness in a sample of primary school children compared to pre-pandemic levels. This increase, however, was not found in infant school children [7].

There are very few studies which have asked the children themselves how they felt during confinement. The results of a study in which Spanish children aged 8 to 17 years completed a questionnaire suggest an ambivalent emotional reaction [8]. Out of the sample, 61% reported boredom, 36% worry, 28% sadness and 16% fear. However, 45% of the sample also expressed happiness at being able to spend more time with their family, 23% at having more time to play and 8% at having more free time. Similar studies in European countries, such as Italy and the United Kingdom, using other report methods, have also found that the emotion most mentioned by parents to describe the state of their children during confinement was boredom [9,10]. In a different study, parents of Spanish children aged between 2 and 14 years also reported emotional ambivalence in children [11]. Thus, parents reported that their children manifested happiness for being at home with their family for longer (84%), while 70% also said they were more highly-strung, 74% that they were more short-tempered and 55% were sadder. Despite these results coinciding both in the self-report and the other-report, it is important to note the limited number of works using assessments completed by the children themselves.

This emotional ambivalence in children as a result of confinement is interesting. It is possible that the complete pause in activity created a greater feeling of calm and wellbeing among the children, and they were able to enjoy their time playing and being with their families. The school environment provides elements for personal and social growth and represents a context of prevention and compensation for children from less favourable environments, but it may also be a space that generates anxiety [12].

### 1.2. Anxiety in the School Context

Anxiety has been defined as an adaptive reaction to situations that represent or are interpreted as a threat or danger for an individual’s integrity or safety [13,14]. Clinical diagnostic systems, such as DSM and ICD, collect and classify the criteria that define anxiety as a disorder [15,16]. Some of these disorders are specific to childhood, such as separation stress and school phobia. Although prevalence varies greatly depending on instruments and contexts, in Spain it is estimated that around 5% of children aged 8 to 12 years may suffer from an anxiety disorder [17]. Previous studies have shown that temperament, parenting conditions, and contextual factors greatly influence the appearance of anxiety disorders [18]. The conceptual delimitation of anxiety exceeds the objectives of this study and for space reasons we cannot enter its discussion. Regarding evaluation, anxiety can be measured through its cognitive (i.e., rumination, worry), behavioral (i.e., nightmares, anxiety or panic attacks), emotional (i.e., feeling scared or being fearful) and physiological components (i.e., palpitations, sweating) [19] (Huberty, 2012). Most of the scales refer to these components for evaluation of anxiety levels in children.

Many studies have tried to identify the contextual factors related to anxiety and depression among primary education school children through scales assessing emotional and conduct problems. The results show that in the school environment, academic demands are associated more directly with states of anxiety, while social relations are associated more with states of depression among the child population [20]. Likewise, other studies note that the start of schooling, the transitions between the different stages of education, assessments and academic performance also represent stressors for the school population [21,22].

Moreover, numerous studies have examined in depth the differences in school anxiety depending on age and gender. Many claims that there are gender differences, with higher levels of school anxiety always among girls than boys, regardless of their age [23,24,25,26]. Other studies have found differences according to age, indicating that anxiety increases among older children and that 11–12 is the age with the highest rates of anxiety [27,28,29,30,31]. In this respect, one of these studies found that among a sample of 758 students studying in the 3rd to 6th year of primary education, levels of anxiety increased significantly with age, and that girls showed higher levels of anxiety than boys [32]. Along the same lines, another research detected higher levels of school anxiety among children in the 6th year (for the Academic Stress factor, i.e., exams, rates, etc.) and in the 3rd and 5th year (for the Physiological Stress factor, i.e., headaches, stomach pain, etc.) [33].

Two conclusions can be drawn from all this previous research. First, confinement resulted in situations of psychological unease among children (more difficulties in self-regulation, distraction, sleep problems and lack of motivation to study)—but at the same time, it may have generated situations of wellbeing. Second, there is a close relationship between anxiety and the school environment, above all for older school students and for girls. Given these two elements, the purpose of this study was to explore whether the impact of confinement and the return to school has led to a change in the levels of anxiety in a sample of 215 children in primary education using a self-report measure of anxiety.

#### The Current Study

This study came about unexpectedly. In March 2020, COVID-19 was declared a pandemic, and Madrid experienced a strict confinement lasting six weeks, leading to a situation of total home reclusion for children. Just before the start of the pandemic, from December to February, our research team had been carrying out an extensive assessment of the children who participated in this study, in order to implement a school intervention program. The data we collected at that time gave us an indication of the psychological state of the children just before the start of the pandemic. Based on pre-pandemic evaluations, children and their families completed questionnaires during confinement and one year after the start of the pandemic.

In this context, one of the scales that interests us most is anxiety. This work therefore compares the levels of anxiety in children before the start of the pandemic (time-point 1—T1) with their levels of anxiety one year later (time-point 3—T3) through a self-reporting measure. Moreover, we compare these two time-point measures with an intermediary one taken during the confinement period (time-point 2—T2), in which a subgroup of children answered the same anxiety scale.

In accordance with prior studies, we expected a reduction in the levels of anxiety during confinement and, as a result of having resumed school activity, a subsequent increase to levels similar to those expressed before the start of the pandemic. We also expected an age and gender effect, with the older children in our sample, and in particular girls, being those expressing the highest levels of anxiety.

## 2. Materials and Methods

### 2.1. Participants

A convenience sample of 215 children (112 girls) aged 6 to 11 years took part in this study (70 to 142 months M = 102.4, SD = 20.20). However, the total number of participants tested in the two periods was somewhat lower (206 in T1 and 205 in T3) because some children did not complete the scale at one of the time-points. At T1, 76 children were in the 1st year of primary school (35%, M = 79.34 months, SD = 3.86), 70 were in the 3rd year of primary school (33%, *M* = 103.08, *SD* = 4.66) and 69 were in the 5th year of primary school (32% M = 127.37, SD = 4.87). At T2 (during confinement), a subgroup of 66 children (41 girls) aged 75 to 144 months (M = 104.93, SD = 18.65) took part. At T3, the participants were between 84 and 153 months old (M = 113.5, SD = 19.9) and in the 2nd, 4th and 6th years of primary school. Table 1 describes the N for each time-point. The children attended two public schools of an urban area of Northwest Madrid and lived in middle and upper-middle class zones.

### 2.2. Procedure

We used an ex post facto longitudinal design for this study. The ethical committee of the faculty approved this research. The schools were selected by convenience for being located in middle class urban areas and for having shown interest in participating in the study. The research team contacted the school psychologist to inform them of the objectives of the study and to explore the possible interest of the school in participating. The school psychologist transmitted the information to the principals. Finally, among the interested schools, two were selected due to the similar socioeconomic characteristics of the population. Likewise, the classes whose teachers agreed to participate in the study were selected. The research team informed the principals and the families about the purpose of the study and parents signed the consent forms. This research followed the protocols for ethical procedures that regulate research in our country.

All the children completed the items on the anxiety scale from the SENA survey (System of Evaluation of Children and Adolescents [34]) twice. T1 was just before the start of the pandemic (from December 2019 to February 2020) and T3 was one year later (from December 2020 to February 2021). For T1 and T3, children completed the questionnaire at school and during school hours. The children went in groups of 10 to the computer room, where they filled out the questionnaire online. Members of the research team, specially trained, helped and supervised the children. For T2, a subgroup of children filled in the same questionnaire after the six strict weeks of confinement in force during the months of March and April 2020 (T2). On this occasion, we included a question allowing the children to comment on their situation and their general state. The question we included read: “Finally, if you want to tell us something about your experience during these days at home, write it here.” For T2. children completed the questionnaire at home with the help and supervision of their parents. Parents received instructions and a link to the questionnaire.

### 2.3. Instruments

The SENA questionnaire, which has been validated and standardized, offers a wide-ranging assessment of the emotional and conduct problems of children aged from 3 to 18 years. The assessment we carried out was broader (44 items), but in this article we only include the results of the anxiety scale. The anxiety scale used in this study is related to generalized anxiety and does not include aspects of social anxiety, somatic complaints, phobia or obsessive–compulsive disorders. The SENA has specific scales for other anxiety disorders that are not included in this study. This scale differs slightly according to age. For children aged 6 and 7 years, the scale consists of 11 items (for example, “I am very afraid of some things”, “I get worried or downcast a lot” and “I have nightmares”). For children of 8 years old and over, the scale is composed of 9 items (including “My problems make me anxious or downcast,” “I’m nervous” and “I think a lot about things”). The score for each item ranges from 1 (never or hardly ever) to 5 (always or almost always). Lower scores indicate a lack of problems and scores of 3 or more indicate the presence of some type of problem. Acceptable reliability was obtained for all three times and for both age groups (Young group: T1 ∝ = 0.71; T2 ∝ = 0.75; T3 ∝ = 0.79. Older group: T1 ∝ = 0.71; T2 ∝ = 0.78; T3 ∝ = 0.85).

As well as the responses to the items on the anxiety scale, we analyzed the answers of children to the final question on their state during the period of confinement. These answers were grouped into 3 categories (positive, negative and ambivalent answer) according to the emotions mentioned by the children. The positive answers only referred to positive emotional states (i.e., “I’m happy at home, I feel very well because I have time to play”); the negative answers only included negative emotional states (i.e., I’m nervous, I feel like a prisoner and I want this to end”); finally, the ambivalent answers mixed positive and negative emotional states (i.e., “I’m happy at home but I miss my friends and I want to see them”). The first author codified all the answers. A second blind codifier codified 50% of the answers. There was a high level of agreement (87%). The disagreements were resolved by consensus.

Statistical Analyses. The scores obtained were analyzed to check the normal distribution. The results indicated a normal distribution at each time-point (Kolmogorov–Smirnov: all *ps* > 0.20). A repeated measures analysis for each age group was performed separately given the configuration of the scale. Two different analyses were used considering gender differences. Student’s t tests were used when no gender differences were found. An ANCOVA was performed when there were gender differences. Additionally, given that the data collected from the sample did not necessarily coincide at all three time-points and that the sample size would be greatly reduced, we performed a comparison between points T1–T2 and between T2–T3 to try and keep as many participants in the comparison as possible.

## 3. Results

Table 1 shows the scores obtained in the three time-points in which the measures were taken, by age. The age groups have been divided into two: younger (6–7 years of age) and older (8–11 years of age), in accordance with different SENA scales.

Age and gender differences. Preliminary analyses did not indicate differences in anxiety among the age groups, either in T1 or T2 (all the *p* values > 0.20). In T3, a univariate test revealed significant differences between the younger and older children (*F*(2, 65) = 1.64, *p* = 0.04, $\eta^{2}\;$= 0.8) with a mean size effect. The differences according to gender were also significant. Table 2 shows the scores according to age and gender at each time-point. At T1. the comparisons show statistically significant differences between girls and boys only in the 6-year-old group with a mean size effect (*M_Boy_* = 2.32, *SD* = 0.62; *M_Girl_* = 2.62, *SD* = 0.61) *F* (1.72) = 4.47, *p* = 0.04, *d* = 0.48). No significant gender differences were found for older children at either time (all the *p*-values > 0.14).

### 3.1. Comparisons of Child Anxiety over Time

To compare the average mean scores of children of 6 years of age in T1 and T3, we executed a repeated measures ANCOVA with gender as a co-variable. On introducing this co-variable, the differences were not significant (*F*(1, 66) = 0.10, *p* = 0.75). For the group of older children (without gender differences), we compared T1 and T3 using a Student’s t-test of repeated measures (with an alpha = 0.01 to avoid type I Errors). The results indicated that the reduction in T3 was statistically significant (*M_T1_* = 2.31, *SD* = 0.68; *M_T3_* = 2.15, *SD* = 0.69; *t* (128) = 2.62, *p* = 0.01), but with a small effect size (*d* = 0.24).

### 3.2. Comparisons with the Intermediate Measure during Confinement (T2)

As we have already mentioned, a small sample of participants (N = 66) completed the questionnaire on anxiety during the confinement. Preliminary analyses indicated that there were no gender differences in T2 (all the *p* > 0.25). No statistically significant differences were found in the group of younger children between T1 and T2 (T1–T2: *t* (23) = 0.30; *p* = 0.77), or between T2 and T3 (T2–T3: *t* (22) = 1.04, *p* = 0.31). In contrast, for the older group, the decline in scores between T1 and T2 did show statistically significant results (*t* (40) = 2.15; *p* = 0.04, *d* = 0.37). The difference between the scores in T2 and T3 was not significant (*t* (40) = –0.49; *p* = 0.63). To sum up, only in the older group did the score decrease significantly between T1–T2.

### 3.3. Responses to Open Question

The answers given by the children to the open question (N = 36) were classified into three groups according to whether the answers were only positive (“I’m fine”, “I have a great time”), only negative (“I want this to end”, “I feel locked in at home”) or ambivalent (“It’s an experience when I’m sad and happy at the same time. The sad part is because I don’t see my friends, and the happy is because I’m with my family a lot”, “I spend more time with my mother and I’m happy for that, but I also want school to open now”). The overall answers of the children were distributed in similar frequencies among the three categories: 12 gave only positive answers, 12 gave only negative answers and a further 12 gave ambivalent answers. To explore the responses more closely, and given that the children could be referring to different aspects of their reality to describe the situation, we decided to count each argument separately. Table 3 shows the frequencies with which we found each of the arguments alluded to by the children.

As can be seen in Table 3, the argument most often used by the children to describe their situation was that being at home and spending time with their family produced a feeling of wellbeing. The next most mentioned aspect was the impossibility of seeing their friends. A very similar number mentioned boredom. Most of the ambivalent arguments combined two or three of these factors, so most of the children either explained their situation only positively or described their family situation positively, but also alluded to the fact that they missed their friends or were bored. Some explanations were particularly revealing in this respect. For example, a 6-year-old boy said “Great, but I’d also like to see my friends”; and an 8-year-old girl said “well, I feel as if I were locked in a cage [...] but at the same time I feel very good because I’m learning to be with my mother more.” Finally, a 6-year-old girl gave a particularly empathetic and moving answer that we have not included in the above classification, but that we nevertheless want to quote: “The only thing I think about a lot in this confinement is the Three Kings and Santa Claus. I’m afraid that the Three Kings and Santa Claus could be infected with the coronavirus.”

## 4. Discussion

The aim of this work was to explore whether the pandemic had affected the anxiety levels of a sample of primary school children. We have also checked these levels with an intermediate measure taken during the confinement. This study has the strength of having a prior measure taken very close to the time of the start of the health crisis, which allows us to make some interesting comparisons. As we already mentioned, the levels of anxiety did not reach clinical significance at any of the three time-points. The results have shown that in comparison with the months before the start of the health crisis, the period of confinement led to a reduction of anxiety levels—significantly in the case of older children. A year later we found lower levels than those before the pandemic—significantly lower in the case of older children, although with a small effect size. Considering our expectations, one year after the beginning of the pandemic we did not find an increase to similar or higher levels than those expressed before the start of the pandemic. Additionally, we did not find higher anxiety levels in older girls. In sum, our results suggest a decrease in anxiety levels and an age effect.

These results are somewhat contradictory with prior studies, which showed an increase in anxiety levels as an effect of the pandemic and confinement [1,2,3]. On the contrary, our results show that confinement led to a reduction in anxiety and that the return to school in the current pandemic conditions has not increased children’s anxiety levels. The fall in the levels of anxiety during confinement may be related to the findings of studies that consider the school context to be a stress factor [21,22,23]. In this respect, the lack of school could have led to this reduction. In line with previous studies, our results also find that this reduction affects older children more [30,31,35]. However, the maintenance of lower levels of anxiety a year later may be due to the influence of other factors. Current life during the pandemic, which to a large extent restricts social and extra-curricular life for children, could be having an effect on this reduction. Our results are partially in line with previous studies regarding gender [36]. Among younger children, girls have significantly greater levels of anxiety and the highest score of all the sample. Although this difference was not found in the older group, it is worth noting that the level of anxiety of older girls during confinement was the lowest of all the sample and time-points.

The qualitative results showed that the explanation most commonly given by the children about their situation during confinement was related to feelings of wellbeing, due to the possibility of spending more time with the family. In addition, some children mentioned missing their friends and being bored, but very few referred to anxiety or edginess as a consequence of confinement. Moreover, analyzed globally, the children’s answers were mainly positive or ambivalent, but not negative. This result coincides with some previous work, in which children also mentioned feeling ambivalent and bored during the confinement [8,9,10,11].

From the point of view of possible applications, these results may make us reflect on the factors that lead to anxiety in the everyday life of children. Before making these considerations, we recall that the levels of anxiety among the children in our sample were within normal limits (remember that scores of three or more indicate the presence of difficulties) and that their belonging to middle and upper socioeconomic classes may explain part of the results, limiting the ability to generalize these conclusions. Even so, these results could suggest that the academic environment may produce some stress for children, that the opportunity to spend more time with their family contributes to their wellbeing, and that the pace of modern life among the middle and upper classes in the Western world could also be affecting these levels of anxiety. We can also relate these results to some particularly worrying data in Spain. Both the OECD and the WHO have alerted Spain on two occasions; according to an OECD study, Spain is the country that imposes the fifth most hours of homework on children (6.5 h per week) [37]. The WHO found that Spanish students feel pressured by academic tasks and that the percentage of Spanish students who suffer stress from homework is among the highest in Europe (for example, 25% in 11-year-old girls and up to 70% in 15-year-old girls) [38]. As stated by the WHO, this pressure translates into anxiety and different somatic manifestations. In addition, the latest report on child and adolescent mental health in Europe by UNICEF revealed that in 2019, the estimated percentage of mental disorders in Spain was the highest in Europe (20.8%) [39]. According to this report, anxiety and depression constitute 54.8% of mental disorders.

Despite anxiety being an adaptive emotion that prepares the organism to be active and to respond adequately to the demands of the environment and possible negative events [35], some of the scores recorded in the first time-point (T1) were close to the threshold that the scale considers as the start of difficulties (for example, in T1, girls aged 6 years obtained a mean of 2.62 and the boys 8 years a mean of 2.42). Despite these levels not indicating any difficulty, the confinement led to a significant reduction in the case of older children, which could indicate some association with academic demands. It is noteworthy that a stressful situation—one of a potentially dangerous health threat for their loved ones, and of great uncertainty, about which the children were probably aware—nevertheless led to a reduction in anxiety levels. We should also consider that these rates are rather high if we take into account the low age of the children, and the good conditions of life they enjoy. Moreover, we found that the children in the sample did not have family and school problems thanks to a scale filled in by the parents in T1 and T2 (these results are not part of this study) [7]. So, what could the anxiety of these children be due to? Our study does not allow us to give a clear answer to this question, but it suggests that in line with previous studies, and considering some of the results from international organizations on academic pressure in Spain, the school environment and perhaps the demands or pace of modern life might result in certain levels of anxiety appearing as early as 6 years of age—even in the best conditions of life possible. Taking the dynamic multifactorial models that explain anxiety levels through the combination of protective and vulnerability factors at specific moments [35], we could think that confinement has been able to act as a protective factor in our sample. Furthermore, given the socioeconomic and family characteristics of our sample, being at home and spending time with their families during confinement may have been able to act as protective factors. On the other hand, as found in previous studies with Spanish, Italian and Portuguese children, after 8 weeks of confinement the parents’ stress levels did not significantly affect the well-being of the children and, as in our study, the anxiety levels of Spanish children decreased significantly when comparing the second and the eighth weeks of confinement [5]. Finally, we can speculate that one year later, and due to the reduction in daily activity, the consequences of the pandemic might have continued to act, in part, as a protective factor in the face of children’s anxiety levels. From a practical point of view, our results, together with data from international organizations, suggest the need to study in detail the causes of anxiety levels in Spanish children. In particular, it would be necessary to determine if the school context is a stressor for children and, if so, to take appropriate measures. All agents involved in the educational system have an important role in detecting, understanding and reducing children’s anxiety levels. COVID-19 could be, in this sense, a red flag to reflect on the educational system and its consequences on the mental health of children.

This work has some limitations that should be taken into account. First, the size of the sample is limited. In the future, much higher samples of children should be used, and the age groups extended. Moreover, as we have already pointed out, the children belong to the middle and upper-middle classes, and this makes it impossible to generalize the results to children who belong to other socioeconomic and cultural contexts. To understand the factors that generate anxiety in children, studies have to be carried out with children belonging to different social, economic and cultural environments. Finally, the scale applied could be considered limited (11 and 12 items). In this respect, it would be desirable to take complementary quantitative and qualitative measures that would allow us to identify the levels and causes of children’s anxiety in greater detail.

## 5. Conclusions

Overall, this study found that the anxiety levels of a sample of children decreased during confinement and one year after the onset of the pandemic. Comparison with levels prior to the start of the pandemic provided an interesting indication of how these levels have varied. Although the study has certain limitations, it could suggest that in certain circumstances and conditions, some populations have not suffered a negative impact on mental health derived from the health situation generated by the coronavirus pandemic. This decrease in anxiety could serve to reflect on the factors (i.e., social and academic demands) that modulate anxiety in primary school children belonging to some socioeconomical and cultural environments.

## Figures and Tables

**Table 1:** Number of participants (N), mean scores (M) and standard deviations (SD) in anxiety scores for each age group in the three test times (T1, T2 and T3).

	6–7 Years			8–11 Years		
	T1	T2	T3	T1	T2	T3
N	73	24	71	133	42	134
M	2.45	2.33	2.27	2.33	2.05	2.16
SD	0.63	0.64	0.72	0.68	0.69	0.74

**Table 2:** Number of participants (N), mean anxiety scores (M) and standard deviation (SD) for girls and boys in the three measurement times (T1, T2, T3) and the two age groups. The lower rows show the results of the comparison between times.

		6–7 Years			8–11 Years		
		T1	T2	T3	T1	T2	T3
Boys	N	40	14	37	59	11	58
	M	2.32	2.40	2.12	2.42	2.25	2.20
	SD	0.61	0.64	0.62	0.76	0.70	0.62
Girls	N	33	10	34	74	31	76
	M	2.62	2.23	2.43	2.25	1.98	2.14
	SD	0.61	0.66	0.80	0.60	0.68	0.83
T1–T3	*F*(1, 66) = 0.10; *p* = 0.75				*t* (128) = 2.62; *p* = 0.01		
T1–T2	*t* (23) = 0.30; *p* = 0.77				*t* (40) = 2.15; *p* = 0.04		
T2–T3	*t* (22) = 1.04; *p* = 0.31				*t* (40) = –0.49; *p* = 0.63		

**Table 3:** Frequencies of arguments used by children to describe their situation during confinement.

Arguments	Frequencies
I’m fine, I feel great at home, I’m with my family	29
I miss my friends	11
I’m bored	10
I miss school, I want to go to school	8
I want this to end, I want to go out, I feel locked in	5
I’m on edge, I feel bad	2
I feel a little alone	1

## Data Availability

Data are available upon request from corresponding author: Marta Giménez-Dasi. magdasi@ucm.es.

## References

[1] Sprang G., Silman M. (2013). Posttraumatic stress disorder in parents and youth after health-related disasters. Disaster Med. Public Health Prep..

[2] Xie X., Xue Q., Zhou Y., Zhu K., Liu Q., Zhang J., Song R. (2020). Mental health status among children in home confinement during the Coronavirus disease 2019 outbreak in Hubei Province, China. JAMA Pediatr..

[3] Jiao W.Y., Wang L.N., Liu J., Fang S.F., Jiao F.Y., Pettoello-Mantovani M., Somekh E. (2020). Behavioral and emotional disorders in children during the COVID-19 epidemic. J. Pediatr..

[4] Orgilés M., Morales A., Delvecchio E., Mazzeschi C., Espada J.P. (2020). Immediate psychological effects of the COVID-19 quarantine in youth from Italy and Spain. Front. Psychol..

[5] Orgilés M., Francisco R., Delvecchio E., Espada J.P., Mazzeschi C., Pedro M., Morales A. (2021). Psychological Symptoms in Italian, Spanish and Portuguese Youth During the COVID-19 Health Crisis: A Longitudinal Study. Child Psychiatry Hum. Dev..

[6] Erades N., Morales A. (2020). Impacto psicológico del confinamiento por la COVID-19 en niños españoles: Un estudio transversal [Psychological impact of confinement due to COVID-19 in Spanish children: A cross-sectional study]. Rev. de Psicol. Clínica con Niños y Adolesc..

[7] Giménez-Dasí M., Quintanilla L., Lucas-Molina B., Sarmento-Henrique R. (2020). Six Weeks of Confinement: Psychological Effects on a Sample of Children in Early Childhood and Primary Education. Front. Psychol..

[8] Martínez Muñoz M., Rodríguez Pascual I., Velásquez Crespo G. Infancia Confinada: ¿Cómo Viven la Situación de Confinamiento Niñas, Niños y Adolescentes? [Confined Childhood: How Do Boys, Girls and Adolescents Live the Confinement?] 2020. https://infanciaconfinada.com/wp-content/uploads/2020/05/informe-infancia-confinada.pdf.

[9] Liang Z., Delvecchio E., Cheng Y., Mazzeschi C. (2021). Parent and Child’s Negative Emotions during COVID-19: The Moderating Role of Parental Attachment Style. Front. Psychol..

[10] Morgul E., Kallitsoglou A., Essau C. (2021). Psychological effects of the COVID-19 lockdown on children and families in the UK. Rev. de Psicol. Clínica con Niños y Adolesc..

[11] Berasategi N., Idoiaga N., Dosil M., Eiguren A., Pikaza M., Ozamiz N. (2020). Las Voces de Los Niños y de Las Niñas en Situación de Confinamiento Por el COVID-19 [Boy’s and Girl’s Voices in COVID-19 Lockdown].

[12] Downey D., Condron D. (2016). Fifty years since the Coleman report: Rethinking the relationship between schools and inequality. Sociol. Educ..

[13] Bourne E.J. (2000). The Anxiety and Phobia Workbook.

[14] Diez C., Sánchez L., Vallejo J., Gastó J. (2000). Trastornos médicos [Medical disorders]. Trastornos Afectivos: Ansiedad y Depresión [Affective Disorders: Anxiety and Depression].

[15] American Psychiatric Association (2013). Diagnostic and Statistical Manual of Mental Disorders (DSM-V).

[16] World Health Organization (2018). International Classification of Diseases 11th Revision (ICD-11).

[17] Orgilés M., Méndez X., Espada J.P., Carballo J.L., Piqueras J.A. (2012). Síntomas de trastornos de ansiedad en niños y adolescentes: Diferencias en función de la edad y el sexo en una muestra comunitaria [Symptoms of anxiety disorders in children and adolescentes: Differences regarding age and gender in a comunitary sample]. Rev. de Psiquiatr. y Salud Ment..

[18] Hay D. (2019). Emotional Development from Infancy to Adolescence.

[19] Huberty T.J. (2012). Anxiety and Depression in Children and Adolescents: Assessment, Intervention, and Prevention.

[20] Trianes M. (2003). Estrés en la Infancia. Su Prevención y Tratamiento [Stress in Childhood. Prevention and Treatment].

[21] Esparza N., Rodríguez M.C. (2009). Factores contextuales del desarrollo infantil y su relación con los estados de ansiedad y depresión [Contextual factors of child development and their relationship with anxiety and depression states]. Divers. Perspect. Psicol..

[22] Pellicer O., Salvador A., Benet I.A. (2002). Efectos de un estresor académico sobre las respuestas psicológicas e inmunes en jóvenes [Effects of academic stressors on psychological and inmunitary responses in youths]. Psicothema.

[23] Oros L.B., Vogel G.K. (2005). Eventos que generan estrés en la infancia: Diferencias por sexo y edad [Events that generate stress in childhood: Differences by gender and age]. Enfoques.

[24] Brumariu L.E., Kerns K.A. (2010). Parent–Child attachment and internalizing symptoms in childhood and adolescence: A review of empirical findings and future directions. Dev. Psychopathol..

[25] Freudenthaler H.H., Spinath B., Neubauer A.C. (2008). Predicting school achievement in boys and girls. Eur. J. Personal..

[26] Fujita M., Fijiwara J., Maki T., Shibasaki K., Shigeta M., Nii J. (2009). Pediatric chronic daily headache associated with school phobia. Pediatr. Int..

[27] Isolan L., Salum G.A., Osowski A.T., Amaro E., Manfro G.G. (2011). Psychometric properties of the screen for child anxiety related emotional disorders (SCARED) in Brazilian children and adolescents. J. Anxiety Disord..

[28] Burnham J.J., Schaefer B.A., Giesen J. (2006). An empirical taxonomy of youths’ fears: Cluster analysis of the American Fear Survey Schedule. Psychol. Sch..

[29] Fremont W.P. (2003). School refusal in children and adolescents. Am. Fam. Physician.

[30] Gómez-Núñez M.I., Aparicio-Flores M.P., Vicent M., Aparisi D., Fernández-Sogorb A., Inglés C.J. (2017). Diferencias en ansiedad escolar en función del sexo y del curso académico en educación primaria [Differences in school anxiety regarding gender and academic grade in elementary education]. Int. J. Dev. Ed. Psychol..

[31] Heyne D., King N.J., Tonge B.J., Cooper H. (2001). School refusal: Epidemiology and management. Paediatr. Drugs.

[32] Orgilé M., Espada J.P., Méndez X., García-Fernández J.M. (2008). Miedos escolares en hijos de padres divorciados y no divorciados [School fears in children of divorced parents]. Int. J. Clin. Health Psychol..

[33] Pérez García M.A. (2019). *Ansiedad Escolar y su Relación Con Variables Psicoeducativas y de Personalidad* [School Anxiety and Its Relationship with Psychoeducational and Personality Variables]. Ph.D. Thesis.

[34] Fernández-Pinto I., Santamaría P., Sánchez-Sánchez F., Carrasco M.A., Del Barrio V. (2015). SENA. Sistema de Evaluación de Niños y Adolescentes [Evaluation System of Children and Adolescents].

[35] Muris P., Merckelbach H., Gadet B., Moulaert V. (2000). Fears, worries, and scary dreams in 4-to 12-year-old children: Their content, developmental pattern, and origins. J. Clin. Child Psychol..

[36] Muris P., Meesters C., Knoops M. (2005). The relation between gender role orientation and fear and anxiety in non-clinic referred children. J. Clin. Child. Adolesc. Psychol..

[37] Organisation for Economic Co-operation and Development (2014). PISA in Focus.

[38] WHO (2016). Health Behavior in School Age Children (HBSC). International Report from the 2011-2014 Survey.

[39] UNICEF (2021). The State of the World’s Children. On My Mind: Promoting, Protecting and Caring for Children’s Mental Health.

